# Radiotherapy as an antihemorrhagic approach in gastric cancer: the RANTIGA study

**DOI:** 10.1007/s00066-026-02542-z

**Published:** 2026-05-12

**Authors:** Valeria Epifani, Francesco Cellini, Luigina Graziosi, Rita Marina Niespolo, Giampaolo Montesi, Najla Slim, Alessio Gili, Nicola Simoni, Eliana La Rocca, Luca Nicosia, Vincenzo Burgio, Filippo Patti, Alessia Surgo, Carmen Molica, Pierfrancesco Franco, Cynthia Aristei, Marco Lupattelli

**Affiliations:** 1 Radiation Oncology Section, University of Perugia, Perugia, Italy; 2 Fondazione Policlinico Universitario “A. Gemelli” IRCCS, Dipartimento di Diagnostica per Immagini, Radioterapia Oncologica ed Ematologia, Rome, Italy; 3 Section of General and Emergency Surgery, Department of Surgery, Santa Maria della Misericordia Hospital, University of Perugia, Perugia, Italy; 4 Radiation Oncology, Fondazione IRCCS San Gerardo dei Tintori, Monza, Italy; 5 Radiation Oncology Unit, AST Pesaro Urbino, Pesaro, Italy; 6 Radiation Oncology Department, San Raffaele Hospital, Milan, Italy; 7 Department of Life Sciences, Health and Health Professions, Link Campus University, Rome, Italy; 8 Radiotherapy Unit, Azienda Ospedaliera Universitaria, Parma, Italy; 9 Department of Radiation Oncology, Azienda Ospedaliera Universitaria Integrata di Verona, Verona, Italy; 10 Department of Advanced Radiation Oncology, IRCSS Sacro Cuore Don Calabria Hospital, Cancer Care Center, Negrar di Valpolicella, Negrar, Italy; 11 Department of Radiation Oncology, SS. Antonio e Biagio and Cesare Arrigo Hospital, Alessandria, Italy; 12 Department of Radiation Oncology, Fondazione IRCCS Istituto Nazionale dei Tumori, Milan, Italy; 13 Department of Radiation Oncology, “F. Miulli” Regional General Hospital, 70021 Acquaviva delle Fonti, Italy; 14 Medical Oncology, S. Maria Della Misericordia Hospital, Perugia, Italy; 15 Department of Translational Sciences (DIMET), University of Eastern Piedmont, Novara, Italy; 16 Radiation Oncology Section, Department of Medicine and Surgery, University of Perugia and Perugia General Hospital, Perugia, Italy; 17 Università Cattolica del Sacro Cuore, Dipartimento Universitario Diagnostica per immagini, Radioterapia Oncologica ed Ematologia, Rome, Italy; 18 Department of Radiation Oncology, “Maggiore della Carità” University Hospital, Novara, Italy

**Keywords:** Gastric cancer, Bleeding, Hemostatic treatment, Palliative radiotherapy

## Abstract

**Introduction:**

Gastric bleeding is a major symptom of locally advanced gastric cancer and a significant cause of mortality. Management options include surgery, endoscopic interventions, embolization and radiotherapy (RT). Although palliative RT appears effective for hemorrhage control, evidences are limited to underpowered retrospective studies from Asia, with issues of patient heterogeneity and response evaluation criteria. This study is a multicenter retrospective analysis carried out across Italian radiation oncology centers to evaluate real-world outcomes of hemostatic RT in patients with bleeding gastric cancer.

**Methodology:**

Clinical and dosimetric data were retrospectively collected for patients with active bleeding gastric cancer treated across twelve Italian radiation oncology centers. The primary endpoint was to evaluate hemoglobin stabilization or improvement at four weeks post-treatment. Secondary outcomes included treatment parameters, acute toxicity profile and time to rebleeding.

**Results:**

Between January 2018 and October 2024, 100 patients receiving hemostatic RT were collected for the analysis. The median age was 77 years, 68% of cases had advanced disease and 41% of patients were pretreated with chemotherapy. The most frequently administered dose was 39 Gy BED10 (range: 9.6–53.1) with a schedule of 30 Gy in 10 fractions. The primary endpoint was achieved in 95/100 patients (95%) indicating stabilization or improvement in Hb levels without the need for post-treatment transfusions. Only 12 patients experienced acute toxicity, of whom 2 (3.3%) presenting grade 3 nausea. Rebleeding symptoms occurred in 38.5% of cases (median interval of 164 days).

**Conclusion:**

This national, retrospective, multicenter study suggests that palliative gastric RT is a feasible, effective and well-tolerated approach in this cohort of patients with bleeding gastric cancer, providing hemostatic control and stabilizing hemoglobin levels. Prospective trials are warranted to better define the role of RT, particularly in terms of dose and fractionation based on the disease setting and patient characteristics.

## Introduction

Gastric cancer remains the fifth most frequently diagnosed malignancy and the third leading cause of cancer-related mortality worldwide. In Italy, it represents approximately 4% of all cancers, with an estimated 14,500 new cases annually [1]. The standard of care for locally advanced gastric cancer involves surgical resection with perioperative chemotherapy. Radiotherapy (RT) plays a limited role and is typically reserved for postoperative cases with non-radical resection margins or inadequate lymphadenectomy. Moreover, RT in the palliative setting is rarely used, particularly especially in Western countries [2]. A significant proportion of patients present with advanced disease and poor performance status, making them ineligible for curative-intent treatment. In such cases, symptom palliation becomes the primary therapeutic objective. Gastric bleeding is a common and potentially life-threatening manifestation, often requiring prompt haemostatic intervention. Available haemostatic approaches include surgery, endoscopic therapy, interventional radiology techniques and RT [3–8]. No comparative prospective trials have been published to define the most effective treatment, although RT offers a non-invasive, well-tolerated alternative option, particularly suitable for elderly or frail patients given the strong rationale based on its haemostatic effect observed in other settings [9]. However, the current evidence consists largely of retrospective studies from Eastern countries, with substantial heterogeneity in treated patients, RT schedules, response criteria to treatment and outcomes, such that the clinical results may not necessarily be generalizable to the European setting [10–13]. Despite RT appears to be infrequently performed in daily practice, especially in Western countries, reported bleeding control rates range from 50% to 95%, with no consensus on the optimal dose or fractionation [12, 14, 15]. The exact mechanism of RT-induced hemostasis is unclear although acute endothelial damage, thrombosis, capillary necrosis with platelet aggregation and tissue factor release likely play a key role [3, 16]. Some data suggest a dose-response relationship, while others support low-dose and short-course regimens [11, 17]. Furthermore, rebleeding rates vary considerably, reflecting the absence of standardized definitions and assessment criteria [10].

The aim of this national multicentre retrospective study was to evaluate the real-world effectiveness and safety of palliative RT for patients with bleeding gastric cancer.

## Materials and methods

The medical records of patients treated with gastric RT for active bleeding between January 1, 2018, and October 31, 2024, across 12 Italian radiation oncology centres affiliated with the Italian Association of Radiation Oncology—Gastrointestinal Study Group (AIRO-GI) were retrospectively reviewed. The study was approved by the Umbria Ethics Committee (protocol no. 4702/24). Data collection was conducted between January and March 2025. Indications for RT were bleeding-related anemia and symptomatic hemorrhage (hematemesis and/or melena) documented by Hb value and upper GI endoscopy. Patients were eligible for the analysis if they had pathologically confirmed gastric cancer and an estimated life expectancy of at least one month. Exclusion criteria included systemic chemotherapy administered within 15 days prior to the start of RT and any invasive procedures performed within the same time frame. The primary endpoint was stabilization or improvement of haemoglobin (Hb) levels at 4 weeks after RT, defined as a variation within ±0.5 g/dL or an increase in Hb concentration. The need of blood transfusion after RT was considered a treatment failure. Secondary endpoints included time to rebleeding, a descriptive analysis of RT parameters and treatment-related acute toxicity, graded according to CTCAE v5.0. Lastly, we explored whether higher Biologically Effective Dose (BED) was associated with improved hemostatic outcomes. BED, calculated using an α/ß ratio of 10, based on a total dose and fractionation schedule, allows comparison of the biological effect of different dose fractionation regimens, even when fraction sizes vary.

## Statistical analysis

Descriptive statistics were used to summarize patient demographics, treatment characteristics, and clinical outcomes. Continuous variables were reported as means with standard deviations (SDs) and 95% confidence intervals (CIs), while categorical variables were presented as frequencies and percentages. The normality of pre- and post-treatment Hb levels was assessed using the Shapiro-Wilk test. Paired Student’s t‑tests were used to compare Hb values before and four weeks after RT. Outlier detection was performed using the Yeo-Johnson transformation and robust Z‑scores. Data points exceeding ±3 SDs were flagged as outliers but retained in the analysis if their influence on overall estimates was negligible. Univariate logistic regression was used to evaluate associations between clinical and treatment variables—including age, sex, prior treatments, and biologically effective dose (BED) of RT—and the primary endpoint (Hb stabilization or improvement). All variables in univariate analysis were entered into a multivariate logistic regression model to identify independent predictors of the primary outcome. Adjusted odds ratios (aORs) with 95% CIs were reported. Statistical significance was defined as a two-tailed *p*-value < 0.05. All analyses were performed using STATA version 17.0.

## Results

A total of 100 patients were included. Patients’ characteristics are summarized in Table 1. The majority were male (73%), with a median age of 77 years (range: 27–97). Adenocarcinoma was the predominant histological subtype (92%) and about 2/3 of patients (68%) had advanced disease (stage IV). Fatigue and melena were the most frequently reported baseline symptoms. Because of gastric bleeding, 82 patients underwent red blood cell transfusions (median: 2; range 1–14). Forty-one patients had received previous chemotherapy.

**Table 1:** Patients’ characteristics.

Number of Patients	100
*Gender*	
Male	73%
Female	27%
*Median Age*	77 (27–97)
*Histology*	
Adenocarcinoma	92%
Squamous Carcinoma	4%
Others	4%
*Symptoms*	
Hematemesis	9%
Melena	22%
Asthenia	38%
Epigastric Pain	13%
More than one	18%
*Blood transfusions prior to RT*	
Yes	82%
No	18%
*Previous Treatment*	
None	58%
Chemotherapy	42%

**Table 2:** Univariate Logistic Regression Analysis for Hemoglobin Response

Variable	Odds Ratio	95% CI	*p* Value
Age	2.30	0.66–7.96	0.189
Sex (Male vs Female)	0.69	0.20–2.34	0.552
Prior Treatments (Yes vs No)	1.68	0.50–5.70	0.405
Dose (≥ vs < 39 Gy BED10)	0.57	0.19–1.73	0.320

**Table 3:** Multivariate Logistic Regression Analysis for Hemoglobin Response

Variable	Odds Ratio	95% CI	*p* Value
Age	2.10	0.55–8.04	0.281
Sex (Male vs Female)	0.75	0.21–2.60	0.652
Prior Treatments (Yes vs No)	1.55	0.45–5.36	0.488
Dose (≥ vs < 39 Gy BED10)	0.63	0.20–1.98	0.428

**Fig. 1:**
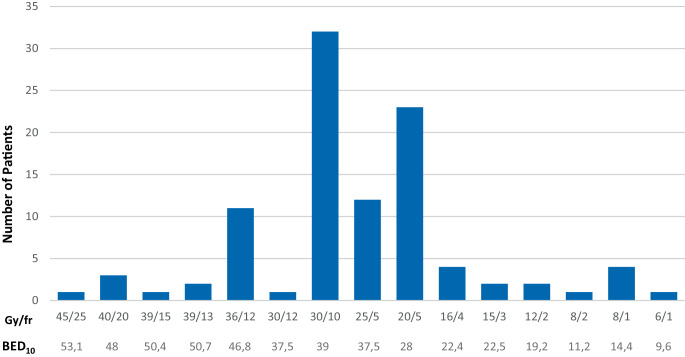
Distribution of dose-fractionation regimens for the treatment of gastric bleeding

RT was mainly delivered using intensity-modulated (IMRT) or volumetric modulated arc technique (VMAT) with a median BED10 of 39 Gy (range: 9.6 Gy–53.1 Gy). The most used dose-fractionation schedules included: 30 Gy in 10 fractions (32%), 20 Gy in 5 fractions (23%), 25 Gy in 5 fractions (12%) and 36 Gy in 12 fractions (11%) (Fig. 1). The clinical target volume (CTV) included the entire stomach or the gross tumor volume (GTV) plus a variable surrounding margin according to the institutional protocols of the participating radiation oncology centres. The median planning target volume (PTV) was 549.4 cc (range: 214–2215 cc).

**Fig. 2:**
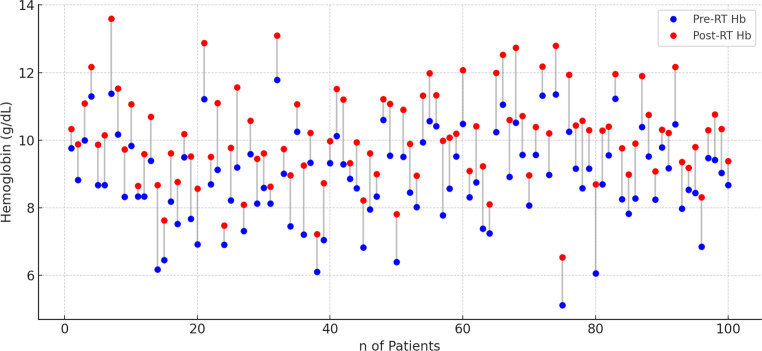
Hb variation pre and post-RT as per single patient

Baseline mean Hb was: 9.03 g/dL (SD 1.49). Mean Hb at 4 weeks post-RT was: 10.29 g/dL (SD 1.47, 95% CI 10.01–10.59). The mean difference between pre- and post-treatment Hb levels was 1.27 g/dL (95% CI 0.94–1.60; *p* < 0.0001), indicating a statistically significant increase. The variation for every patient is shown in Fig. 2.

The primary endpoint was achieved in 95 out of 100 patients (95%), indicating Hb stabilization or improvement without the need for post-treatment transfusions.

Normality of both pre- and post-RT Hb distributions was confirmed using the Shapiro-Wilk test (Hb pre-RT: W = 0.9809, *p* = 0.156; Hb post-RT: W = 0.9796, *p* = 0.123). Outlier analysis identified only two anomalous data points (2%), which did not significantly impact the results, and all patients were retained in the final analysis.

Regarding treatment-related toxicity, 12 patients experienced acute adverse events, with only 2 cases (3.3%) reaching grade 3 severity (nausea). No grade 4 or higher events were reported. Ninety-eight percent of patients successfully completed the scheduled treatment.

Bleeding recurrence was evaluated in 70 patients as we were not able to collect this clinical information for 30 cases. Twenty-seven out of seventy patients (38.5%) experienced bleeding recurrence, with a median bleeding-free interval of 164 days (range: 30–1164 days). One of these patients received embolization while five patients (18.5%) underwent reirradiation. After receiving very heterogeneous RT schedules (40 Gy/20, 30 Gy/10, 30 Gy/10, 12 Gy/2 and 8 Gy/1 fractions), the retreatment RT regimens were 20 Gy/5 fractions in two, 8 Gy/1 fraction in two and 20 Gy/4 fractions in one patient. In these cases, the target volumes included the gross tumour volume plus a variable surrounding margin of 1–2 cm.

Univariate logistic regression analysis for potential predictors of response (age, sex, prior treatments, BED10 radiation dose) did not identify any statistically significant associations. However, age showed a non-significant trend toward predictive value (OR = 2.30, 95% CI: 0.66–7.96, *p* = 0.189). The results of the predictor analyses for hemoglobin response are reported in Tables 2 and 3.

## Discussion

Despite the limitations of this study, the present analysis represents the first Italian multicenter retrospective study demonstrating that palliative RT is an effective and well-tolerated option for the management of active gastrointestinal bleeding in patients with gastric cancer.

Hemostatic efficacy was high, with 95% of patients achieving stabilization or improvement in Hb levels within one month following RT. Data from systematic reviews reported response rates ranging from 50% to 95% [15–18]. Our results compare favorably even with prior prospective studies: Tey et al. [19] reported bleeding control in 80% of patients treated with 36 Gy/12 fractions (BED10 48.6 Gy), while Saito et al. [20], in a multicenter study (2022), documented a response rate of 69% with a median dose of 20 Gy/5 fractions (BED10 28 Gy), and Tanaka et al. [21] obtained hemostatic efficacy in 80% of cases after 20 Gy/5 fractions (BED10 28 Gy).

The radiation regimens employed in our cohort varied considerably, reflecting the heterogeneity reported in previous literature and underscoring the absence of consensus regarding the optimal dose-fractionation schedule. Although 30 Gy in 10 fractions was the most used schedule, various regimens may be considered with fraction doses ranging from 1.8 to 8 Gy and total doses from 6 to 60 Gy, corresponding to BED10 of 7.2–50.8 Gy [11, 15–18]. We did not observe any correlations between clinical response and BED10, consistent with findings by Kawabata et al. [14] and Tey et al. [17], which support the adequacy of low to moderate BED regimens for symptom palliation, although other authors described a stronger correlation with BED10 dose > 30–39 Gy [12, 13, 16, 22]. To date, while there may be no difference between low and high BED regimens in bleeding palliation, local control might be improved with higher BED regimens, as reported by some studies [12, 13, 15, 16, 23–25]. The results of the recently published TOPGEAR trial [26] may support a possible dose-response correlation.

RT was well tolerated even in an elderly population (median age: 74.9 years), with only 3.3% of patients experiencing grade ≥ 3 adverse events, primarily gastrointestinal (e.g., nausea), confirming the favorable safety profile observed in prior studies. An overview of toxicity data published in the literature showed that severe events occurred in a range of 0–29%, but usually less than 10% [15–18]. Our safety profile may be related to the use of intensity-modulated RT rather than 3D conformal RT, as in most published reports. Previous experiences, as well as our study, have reported target areas ranging from partial to the whole stomach [16–18]. Since the best approach is unclear, it seems reasonable to initially include the whole stomach, while for any re-irradiation the field was contoured using clips placed endoscopically near the lesion to reduce treatment-related toxicity [21]. Compliance with treatment was very high, as 98% of patients completed the scheduled RT regimen. Treatment completion rates reported in the literature were also high (> 80%), even in the definitive setting where high doses (BED10 up to 59,47 Gy) were delivered to large volumes. To confirm these data, in the TOPGEAR trial [26], 92% of cases completed the planned RT of 45 Gy/25 fractions (BED10 53 Gy).

In our experience, recurrence of bleeding symptoms occurred in 38.5% of cases, with a median time to rebleeding of approximately 5.5 months. This result is consistent with literature data, which report rebleeding in about 5–60% of patients, with a bleeding-free survival of 1.6–12 months [10], although the contributing factors, including study design, patient characteristics, and RT fractionations, were heterogeneous. A further clinical issue affecting comparison of published data, which may explain the wide range of events, is represented by the definition of rebleeding, which may include a reduction of Hb values below 7 g/dL, the need for blood transfusions, and/or recurrence of clinical symptoms [16–18].

According to our results, five out of 27 patients (18.5%) experiencing rebleeding underwent a second course of RT and achieved hemostatic control. Although retreatment was not among the stated objectives of this retrospective analysis, our data are comparable to those published in the literature. Kawabata et al. [10] and Tanaka et al. [21], following first low-dose irradiation of BED10 28 Gy and 7.2 Gy, obtained hemostasis in 100% and 75% of cases, respectively. However, given the small number of cases, re-irradiation should be carefully evaluated after considering prior treatment, patient clinical condition, and risk factors for severe treatment-related adverse events.

Several general limitations related to the existing literature should be acknowledged. Firstly, data regarding the effectiveness, safety, and tolerability of gastric palliative RT are derived mainly from retrospective Asian studies. In Western countries, gastric palliative RT is not commonly practiced, as European and American guidelines recommend [27, 28]. A systematic review of gastric RT given as a definitive and palliative option [15] showed that among the twenty-one studies collected in the palliative setting, only two were carried out in Western countries [29, 30]. In European countries, very few experiences have been published; a recent survey of UK oesophago-gastric clinical oncologists’ use of gastric RT showed that 93% had prescribed palliative intent RT (dose < 40 Gy) over the preceding 3 years, compared to only 16.7% in the definitive setting (> 40 Gy) [31]. Furthermore, many confounding factors emerge when comparing published data, as response criteria vary across studies, including differences in time points for response evaluation and definitions of bleeding control [16–19].

In addition, several limitations specific to the present study must be considered. The retrospective design inherently limits causal inference and is subject to potential selection and reporting biases. Although relatively large for this clinical setting, the sample size (*N* = 100) remains limited and may reduce the statistical power of subgroup analyses. Moreover, as the study was conducted within a single country, the generalizability of the findings to other European populations or healthcare systems may be limited. Transfusion practices were not standardized, in contrast with the structured approach reported by Lee et al., which included transfusion timing and thresholds relative to RT. Finally, patient-reported outcomes, including health-related quality of life (HRQoL), were not assessed, highlighting a critical area for future prospective investigations—an unmet need also emphasized in the review by Tey et al. [19].

The principal strengths of our study include its national, multicenter design, a relatively large sample size, and a focus on real-world clinical practice, reflecting different institutional protocols and patient populations across 12 Italian centers affiliated with the AIRO GI study group.

In summary, these results advocate for future prospective studies aimed at optimizing dose-fractionation regimens and incorporating patient-centered outcomes to better define the therapeutic role of RT in this palliative setting. Presently, in clinical practice, it may be reasonable to use low BED10 regimens for frail patients with poor performance status, low life expectancy, and high disease burden, while offering high BED10 regimens to patients with a longer life expectancy, for whom improved local control may increase the likelihood of using further therapeutic options.

## Conclusion

This study supports the use of palliative RT as a feasible, effective, and safe intervention for bleeding control in gastric cancer patients. Prospective trials are needed to optimize dose-fractionation regimens according to patient characteristics and life expectancy.

## Data Availability

The data supporting the findings of this study are available from the corresponding author upon reasonable request, subject to institutional approval and ethical/privacy restrictions.
